# Supplementary material for: XPF mediates 3′ flap processing for FEN1-independent Okazaki fragment maturation
Source: Nucleic Acids Res. 2026 May 26;54(10):gkag536. doi: 10.1093/nar/gkag536 (PMC13202174; doi:10.1093/nar/gkag536)
Supplement: gkag536_Supplemental_File [file gkag536_supplemental_file.pdf]

Table S1: Random spore analysis of the SL phenotype of *rad1*Δ *rad27*Δ and suppression of the SL phenotype by *pol3* ITD

Genetic cross	Colony Number (YPD plates)	Colony Number (SC plates)	Observed ratio	Expected Ratio	Viability
*rad1*Δ::Trp1<br>×<br>*rad27*Δ::Ura3	103	*rad1*Δ *rad27*Δ<br>3	0.029	0.25	NO
*rad1*Δ::Trp1<br>×<br>*rad27*Δ::Ura3<br>*pol3* ITD::His3	154	*rad1*Δ *rad27*Δ *pol3* ITD<br>17	0.110	0.125	YES
*rad1*Δ::Trp1<br>×<br>*pol3* ITD::His3	645	*rad1*Δ *pol3* ITD<br>241	0.374	0.25	YES

Table S2: Random spore analysis of the SL phenotype of *mre11*Δ *rad27*Δ and suppression of the SL phenotype by *pol3* ITD

Genetic cross	Colony Number (YPD plates)	Colony Number (SC plates)	Observed ratio	Expected Ratio	Viability
*mre11*Δ::Trp1 × *rad27*Δ::Ura3	961	*mre11*Δ *rad27*Δ 6	0.006	0.25	NO
*mre11*Δ::Trp1 × *rad27*Δ::Ura3 *pol3* ITD::His3	768	*mre11*Δ *rad27*Δ *pol3* ITD 3	0.004	0.125	NO
*mre11*Δ::Trp1 × *pol3* ITD::His3	606	*mre11*Δ *pol3* ITD 174	0.287	0.25	YES

Table S3: Random spore analysis of the SL phenotype of *mus81*Δ *rad27*Δ and suppression of the SL phenotype by *pol3* ITD

Genetic cross	Colony Number (YPD plates)	Colony Number (SC plates)	Observed ratio	Expected Ratio	Viability
*mus81*Δ::Trp1 × *rad27*Δ::Ura3	81	*mus81*Δ *rad27*Δ 2	0.025	0.25	NO
*mus81*Δ::Trp1 × *rad27*Δ::Ura3 *pol3* ITD::His3	400	*mus81*Δ *rad27*Δ *pol3* ITD 61	0.153	0.125	YES
*mus81*Δ::Trp1 × *pol3* ITD::His3	81	*mus81*Δ *pol3* ITD 18	0.222	0.25	YES

Table S4: Random spore analysis of the SL phenotype of *sae2*Δ *rad27*Δ and suppression of the SL phenotype by *pol3* ITD

Genetic cross	Colony Number (YPD plates)	Colony Number (SC plates)	Observed ratio	Expected Ratio	Viability
*sae2*Δ::Trp1 × *rad27*Δ::Ura3		*sae2*Δ *rad27*Δ		0.25	NO
*sae2*Δ::Trp1 × *rad27*Δ::Ura3 *pol3* ITD::His3	530	*sae2*Δ *rad27*Δ *pol3* ITD 3	0.006	0.125	NO
*sae2*Δ::Trp1 × *pol3* ITD::His3		*sae2*Δ *pol3* ITD		0.25	

Table S5: Random spore analysis of the SL phenotype of *sgs1*Δ *rad27*Δ and suppression of the SL phenotype by *pol3* ITD

Genetic cross	Colony Number (YPD plates)	Colony Number (SC plates)	Observed ratio	Expected Ratio	Viability
*sgs1*Δ::Trp1 × *rad27*Δ::Ura3	68	*sgs1*Δ *rad27*Δ 0	0	0.25	NO
*sgs1*Δ::Trp1 × *rad27*Δ::Ura3 *pol3* ITD::His3	38	*sgs1*Δ *rad27*Δ *pol3* ITD 5	0.132	0.125	YES
*sgs1*Δ::Trp1 × *pol3* ITD::His3	68	*sgs1*Δ *pol3* ITD 16	0.235	0.25	YES

Table S6: Random spore analysis of the SL phenotype of *srs2*Δ *rad27*Δ and suppression of the SL phenotype by *pol3* ITD

Genetic cross	Colony Number (YPD plates)	Colony Number (SC plates)	Observed ratio	Expected Ratio	Viability
*srs2*Δ::hph × *rad27*Δ::Leu2	115	*srs2*Δ *rad27*Δ 0	0	0.25	NO
*srs2*Δ::hph × *rad27*Δ::Leu2 *pol3* ITD::His3	223	*srs2*Δ *rad27*Δ *pol3* ITD 7	0.031	0.125	NO
*srs2*Δ::hph × *pol3* ITD::His3	1226	*srs2*Δ *pol3* ITD 254	0.207	0.25	YES

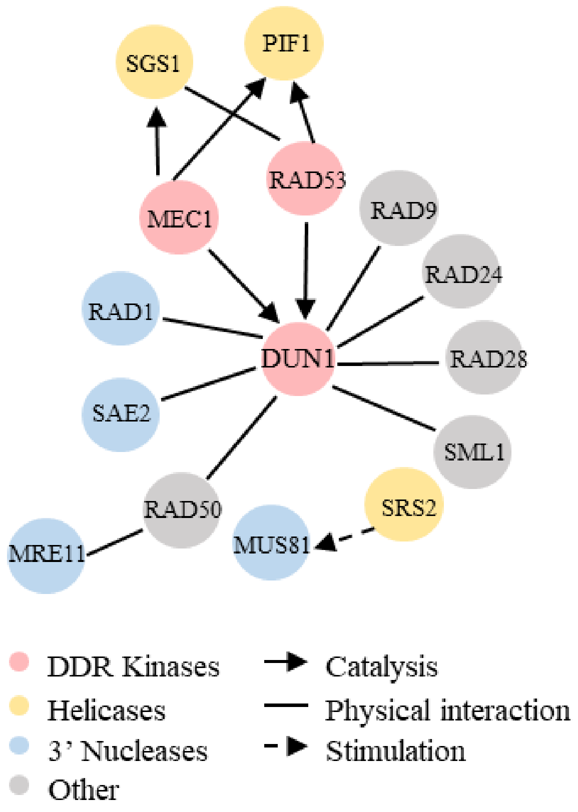

Supplementary Figure S1: **Physical interactions of DNA repair proteins with the Mec1-Rad53-Dun1 axis.** Saccharomyces Genome Database (SGD) was surveyed for DUN1 interaction helicases and nucleases.

A

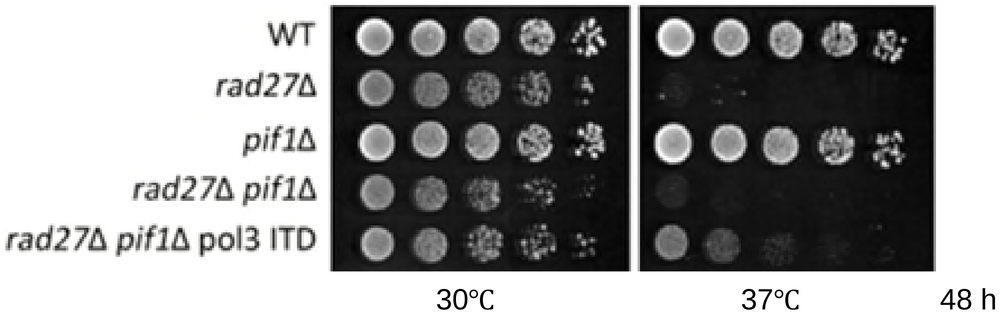

B

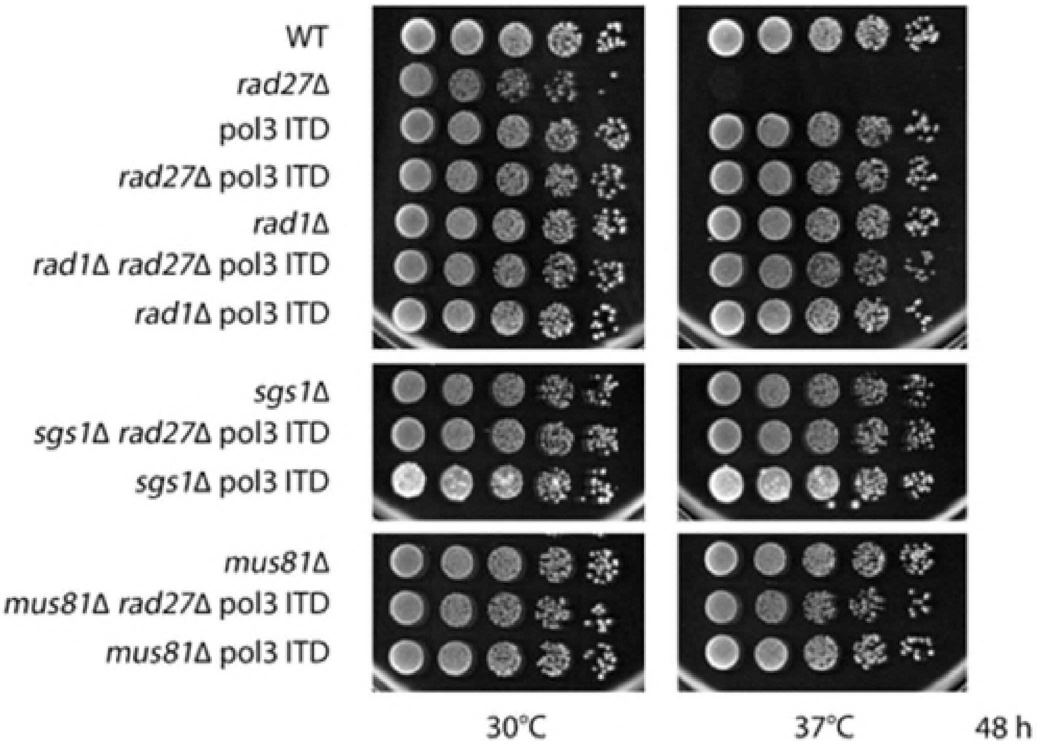

Supplementary Figure S2: Spot assays to verify the viability of yeast cells. (A) Spot assays on WT, *rad27*Δ, *pif1Δ*, *rad27*Δ *pif1Δ, or rad27*Δ *pif1Δ* pol3-ITD at 30℃ (optimal temperature) or 37℃ (restrictive temperature). **(B)** Spot assays on WT, *rad27*Δ, *rad1Δ*, *sgs1Δ, mus81Δ, rad27*Δ *rad1Δ, rad27*Δ *sgs1Δ, rad27*Δ *mus811Δ, or rad27*Δ *rad1Δ pol3-ITD, rad27*Δ *sgs1Δ pol3-ITD, rad27*Δ *mus811Δ pol3-ITD* at 30℃ or 37℃.

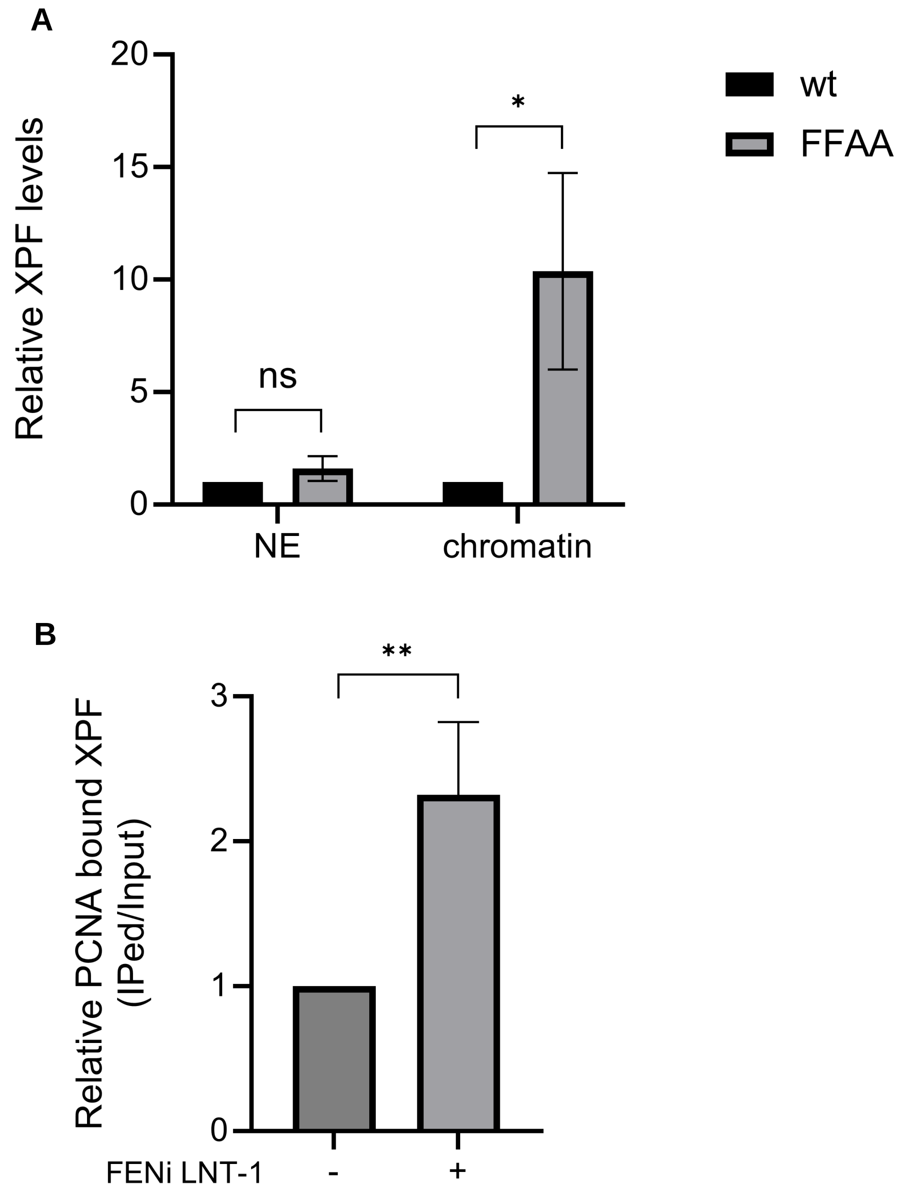

Supplementary Figure S3: Quantification of immunoblot analysis showing increased XPF recruitment upon FEN1 mutant or inhibition. (A) Quantification of XPF protein levels shown in figure1 A. Band intensities were quantified using ImageJ, normalized to Histone H3 or GAPDH, and expressed relative to WT cells. Data represent mean ± SEM from three independent experiments; **(B)** Quantification of XPF protein levels shown in figure1 B. Band intensities were quantified using ImageJ, normalized to input XPF, and expressed relative to IP control group. Data represent mean ± SEM from four independent experiments. *P<0.05, **P < 0.01. P values are calculated by the student's t-test.

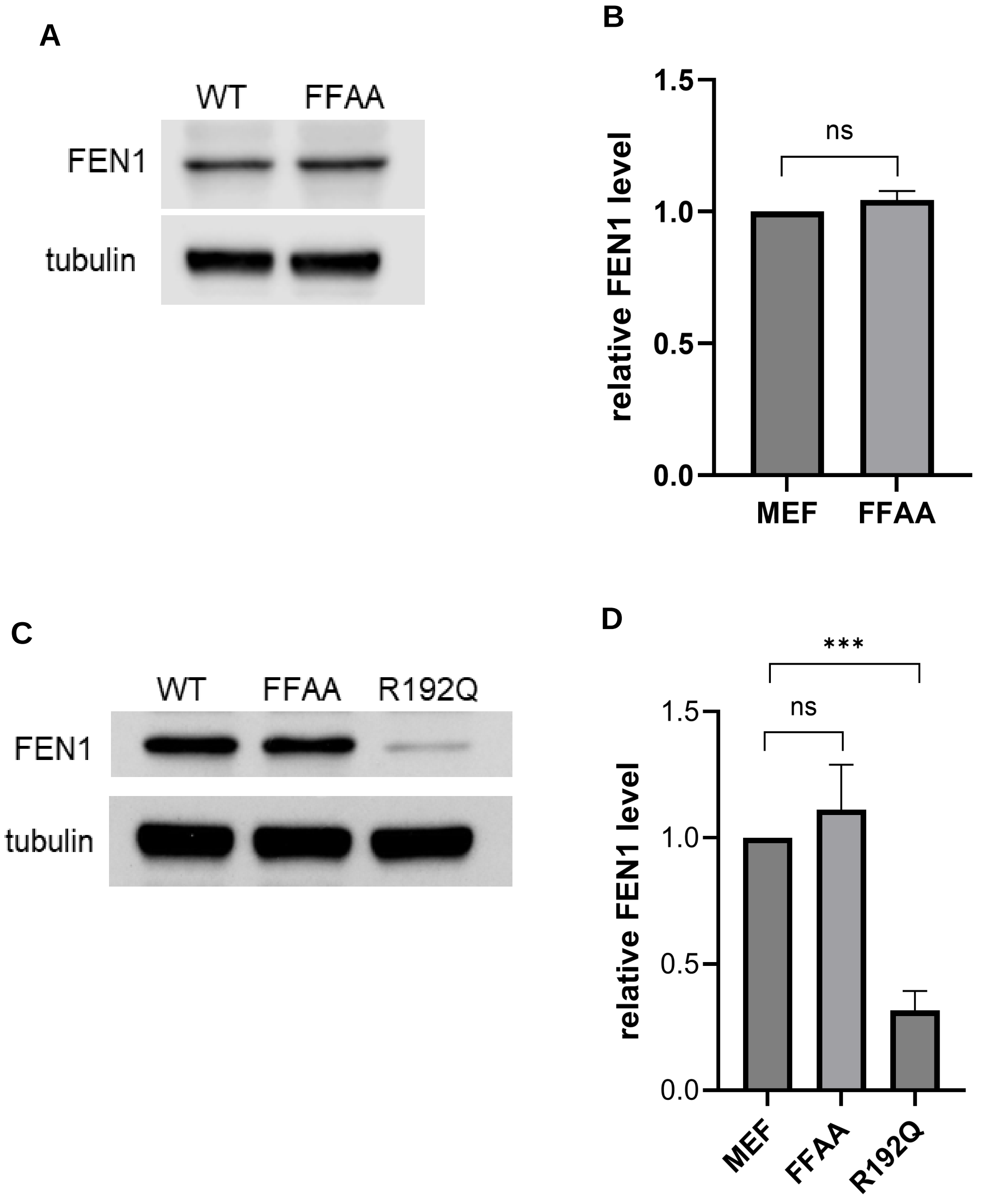

Supplementary Figure S4: FEN1 levels in WT, FFAA or R192Q mutant MEF cell lines. **(A)** Immunoblot analysis of FEN1 expression in WT and FFAA MEFs shows comparable protein levels; **(B)** Quantification of FEN1 protein levels shown in (A). Band intensities were quantified using ImageJ, normalized to Tubulin, and expressed relative to WT cells. Data represent mean ± SEM from three independent experiments; **(C)** Immunoblot analysis of FEN1 expression in WT, FFAA and R192Q MEFs; **(D)** Band intensities were quantified using ImageJ, normalized to Tubulin, and expressed relative to WT cells. Data represent mean ± SEM from three independent experiments. ***$P < 0.001$. P values are calculated by the student's t-test.

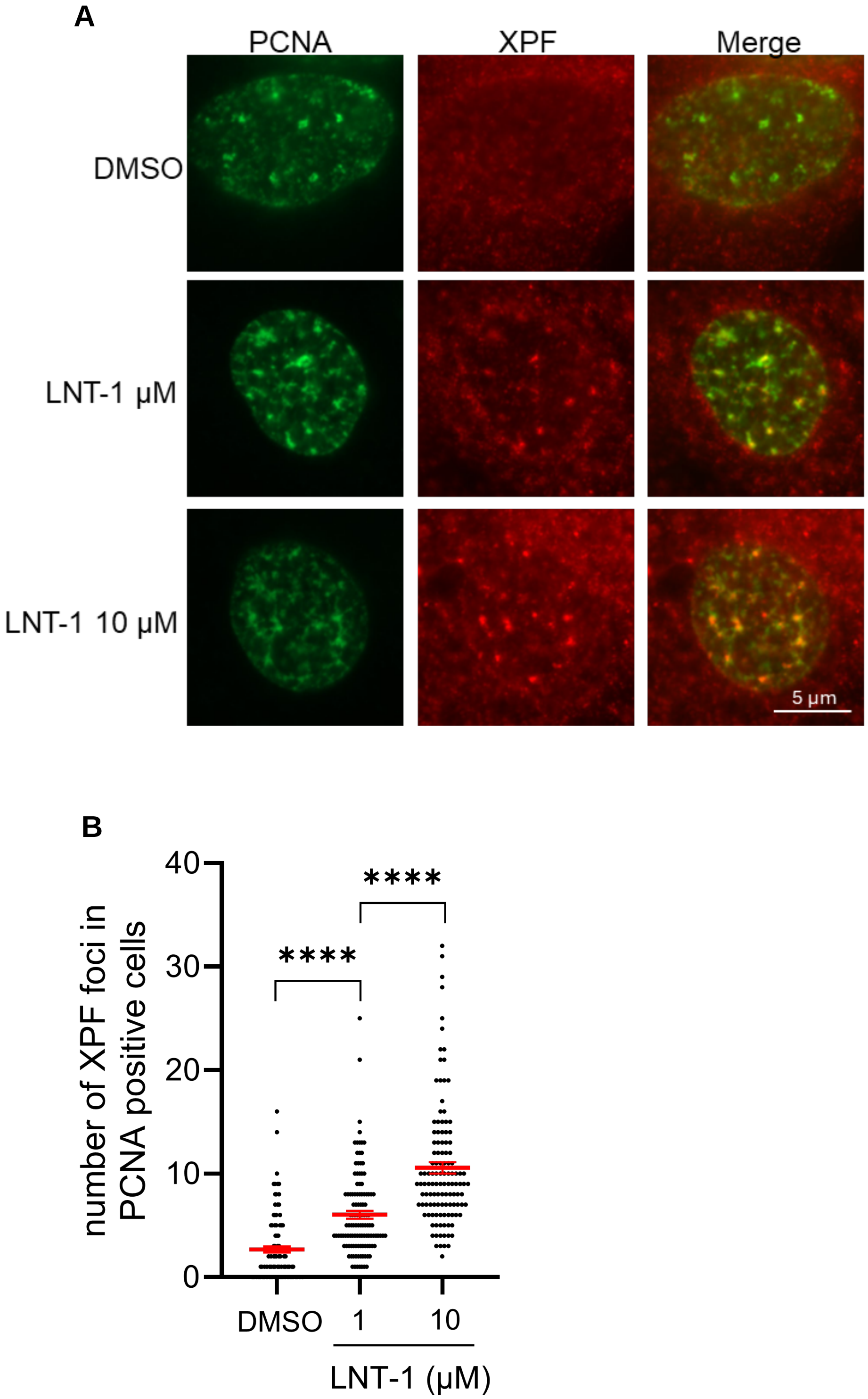

Supplementary Figure S5: co-IF staining of PCNA and XPF in MEFs in the absence or presence of FEN1i. **(A)** Representative microscope images of XPF-PCNA co-IF staining in MEF cells treated with DMSO or FEN1i LNT-1; **(B)** Quantification of the XPF-PCNA co-localized foci number per cell. MEFs were treated with DMSO or FEN1i LNT-1 (1 μM or 10 μM) for 16hr. Data represent mean ± SEM from ≥100 cells per condition. **** $p < 0.0001$. P values were calculated with the Student t-test.

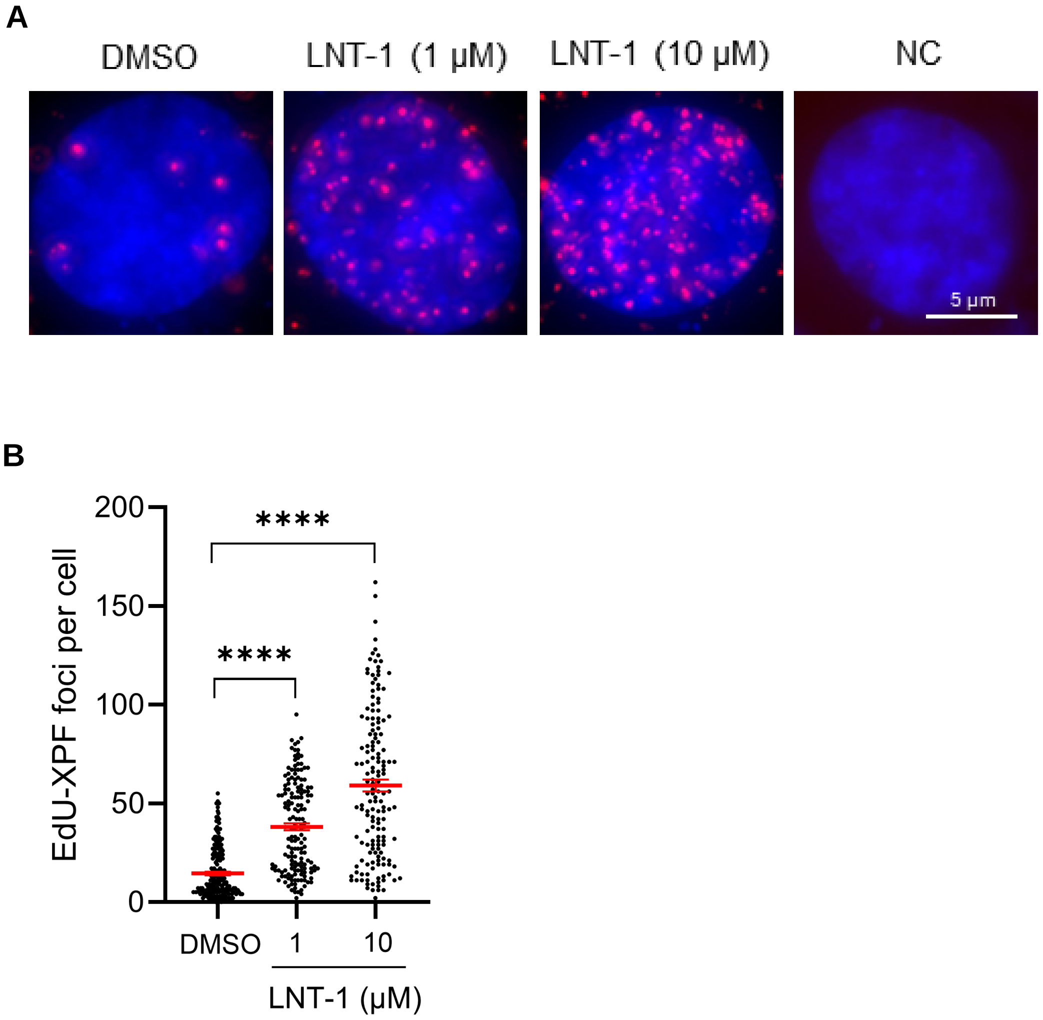

Supplementary Figure S6: EdU-XPF PLA in MDA-MB-231 cells. (A) Representative microscope images of PLA performed in MDA-MB-231 cells treated with DMSO or FEN1i; **(B)** Quantification of the EdU-XPF PLA foci number in MDA-MB-231 cells. Cells were treated with DMSO or LNT1 (1and 10 μM) for 16hr and labeled with10 μM EdU before harvest. Single-antibody negative controls were performed in LNT-1 (10 μM) treatment cells and showed minimal background signal. Data represent mean ± SEM from ≥100 cells per condition. ****P < 0.0001. P values are calculated by the student's t-test.

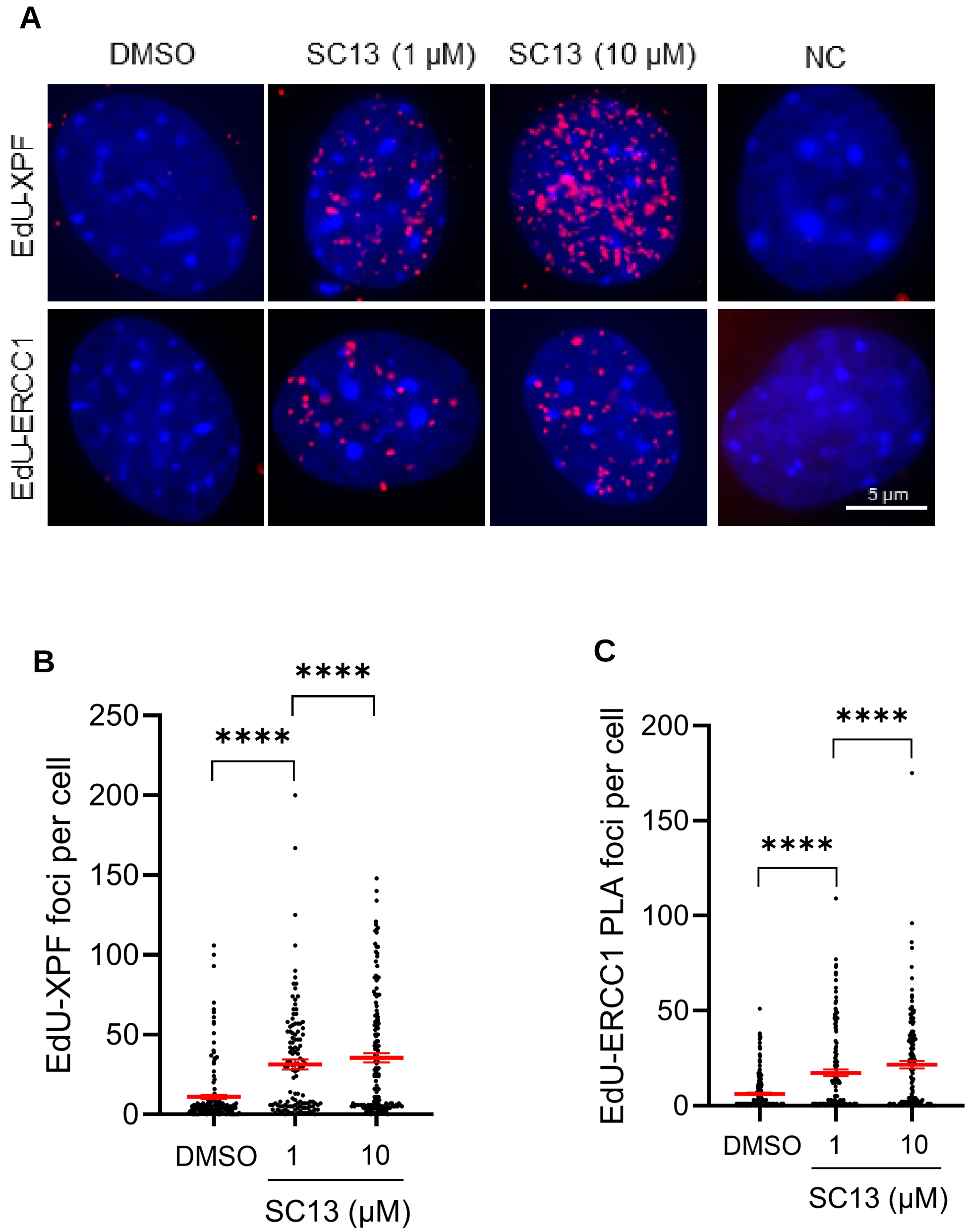

Supplementary Figure S7: Validation of EdU-ERCC4/ERCC1 PLA using an independent FEN1 inhibitor. (A) Representative microscope images of PLA performed in MEF cells treated with DMSO or FEN1i SC13; Quantification of the EdU-ERCC4 **(B)** and EDU-ERCC1 **(C)** PLA foci number in MEF cells. Cells were treated with DMSO or FEN1i SC13 (1 and 10 μM) for 16hr and labeled with10 μM EdU before harvest. Single-antibody negative controls were performed in SC13 (10 μM) treatment cells and showed minimal background signal. Data represent mean ± SEM from ≥100 cells per condition. ****P < 0.0001. p values are calculated by the student's t-test.

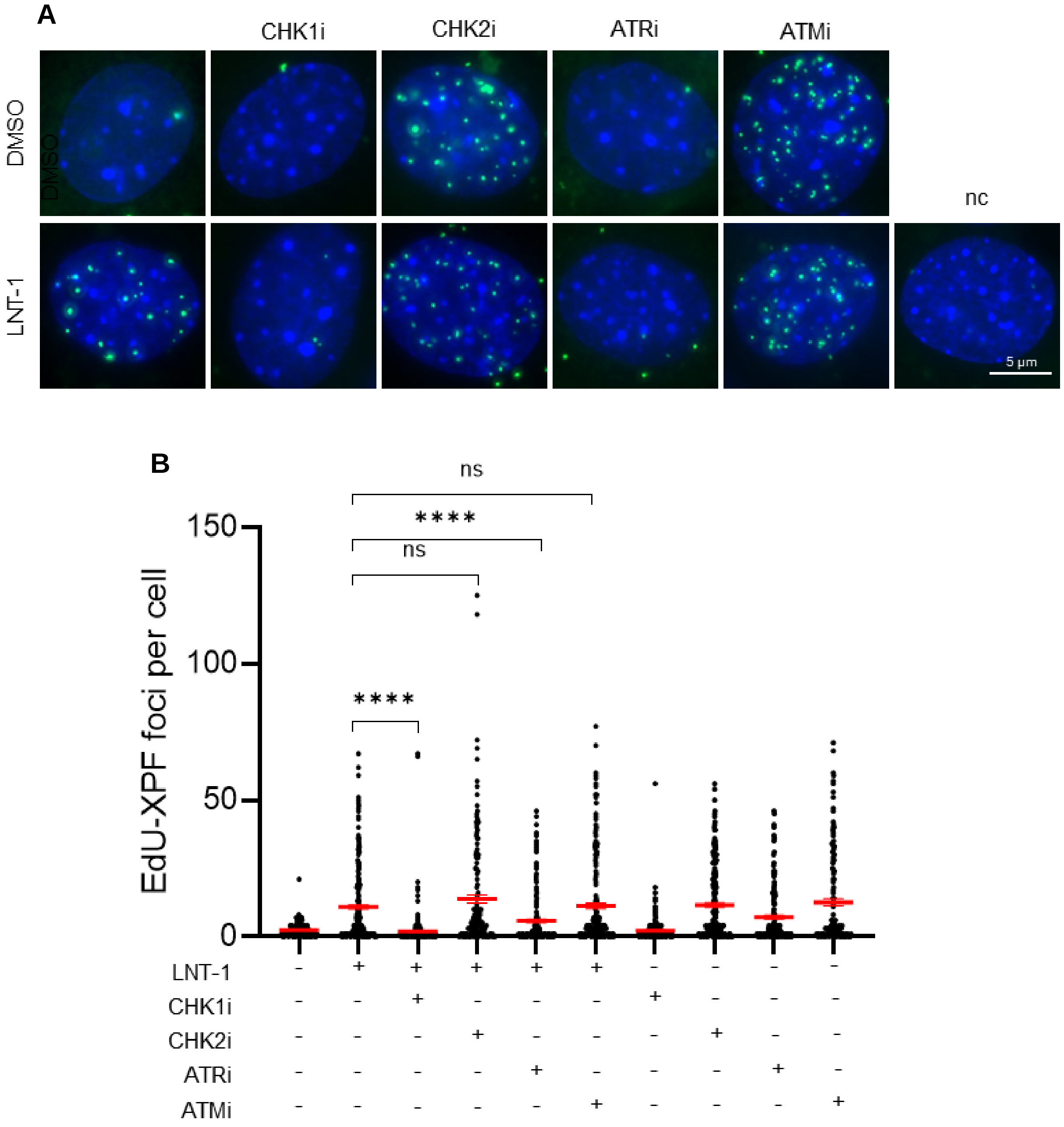

Supplementary Figure S8: Effect of DDR kinase inhibitors on EdU-XPF PLA signals induced by FEN1 inhibition. (A) Representative microscope images of PLA performed in MEF cells treated with DMSO, FEN1i LNT-1,CHK1i, CHK2i, ATRi or ATMi; **(B)** Quantification of the EdU-XPF PLA foci number in MEF cells. Cells were treated with DMSO or inhibitor(10 μM) for 16hr and labeled with10 μM EdU before harvest. Data represent mean ± SEM from ≥100 cells per condition. ****P < 0.0001. p values were calculated by the student t-test.

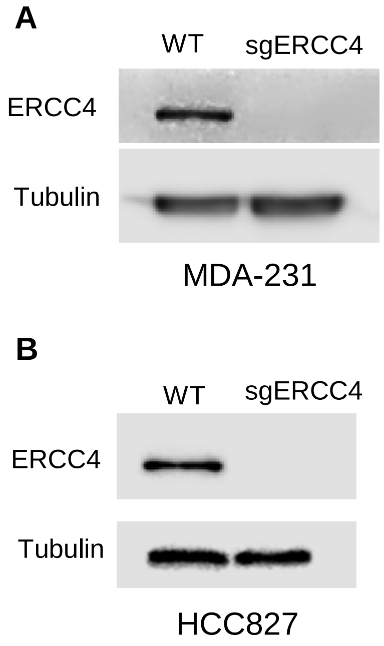

Supplementary Figure S9: Validation of ERCC4/XPF knockout in MDA-231 and HCC827 cells. (A) Immunoblot analysis of ERCC4/XPF protein levels in wild-type (WT) and sgERCC4 MDA-MB-231 cells. Tubulin was used as a loading control; **(B)** Immunoblot analysis of ERCC4/XPF protein levels in wild-type (WT) and sgERCC4 HCC827 cells. Tubulin was used as a loading control.

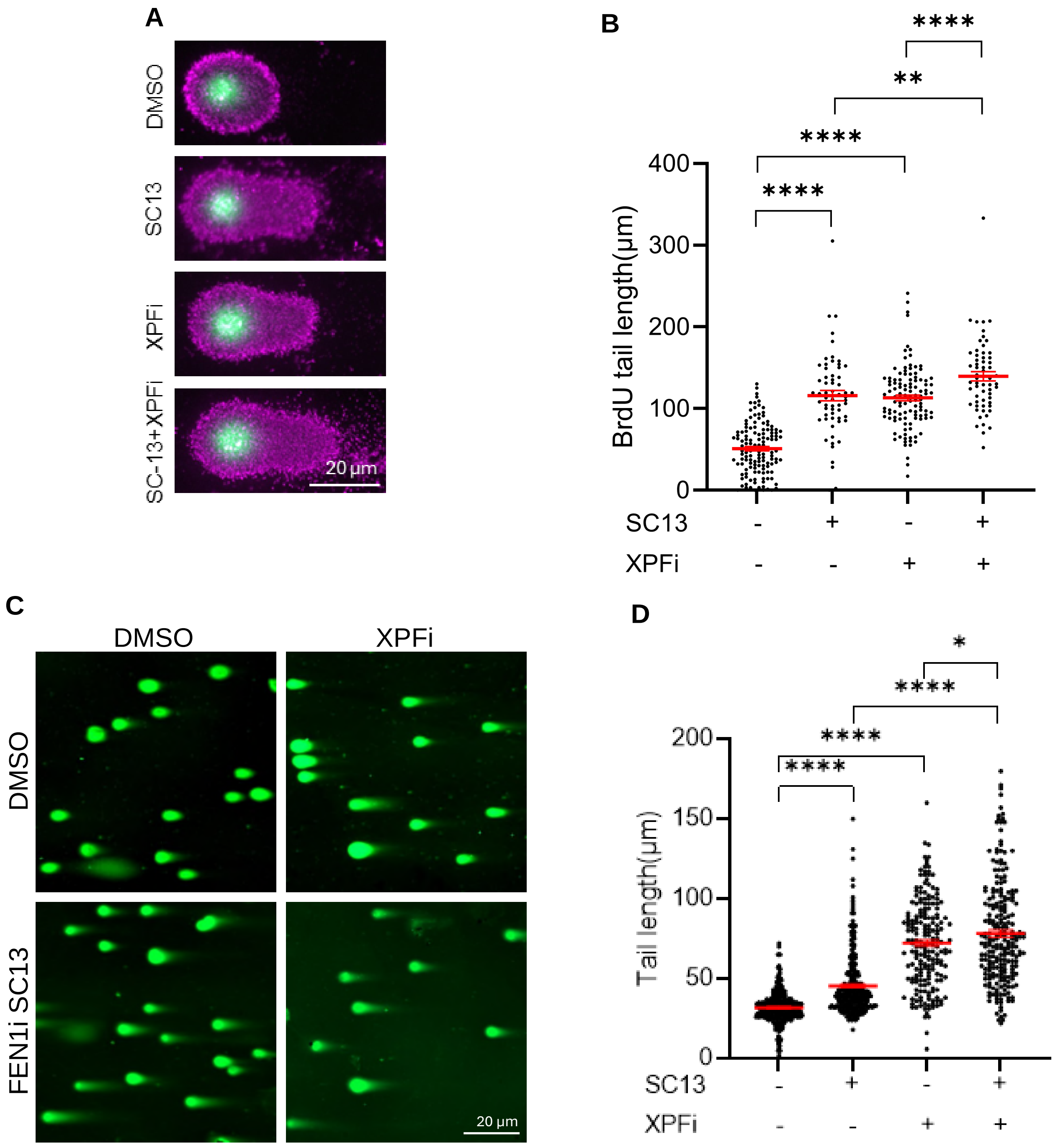

Supplementary Figure S10: BrdU comet assay and Neutral comet assay performed in DMSO/SC13 treatment MDA-MB-231 cells. (A) and **(B)** BrdU comet assay performed in MDA-MB-231 cells. Panel (A) shows representative microscope images of BrdU Comet assay in MDA-MB-231 cells treated with DMSO/ FEN1i SC13/ XPFi. Panel (B) shows quantification of BrdU tail length per cell in BrdU comet assay. Cells were treated with DMSO/ SC13 (10 μM)/ XPFi (10 μM) for 16hr and labeled with 20 μM BrdU for 20min before harvest. Data represents mean ± SEM from ≥50 cells per condition. **P < 0.01, ****P < 0.0001. p values were calculated by the student t-test; **(C)** and **(D)** Neutral Comet assay performed in MDA-MB-231 cells. Panel (C) shows representative microscope images of neutral Comet assay in MDA-MB-231 cells treated with DMSO/ FEN1i SC13 / XPFi, and panel (D) is quantification of DNA tail length per cell in neutral Comet assay. Cells were treated with DMSO/ SC13(10 μM)/ XPFi (10 μM) for 16hr. Data represents mean ± SEM from ≥100 cells per condition. *P < 0.05, ****P < 0.0001. p values were calculated by the student t-test.

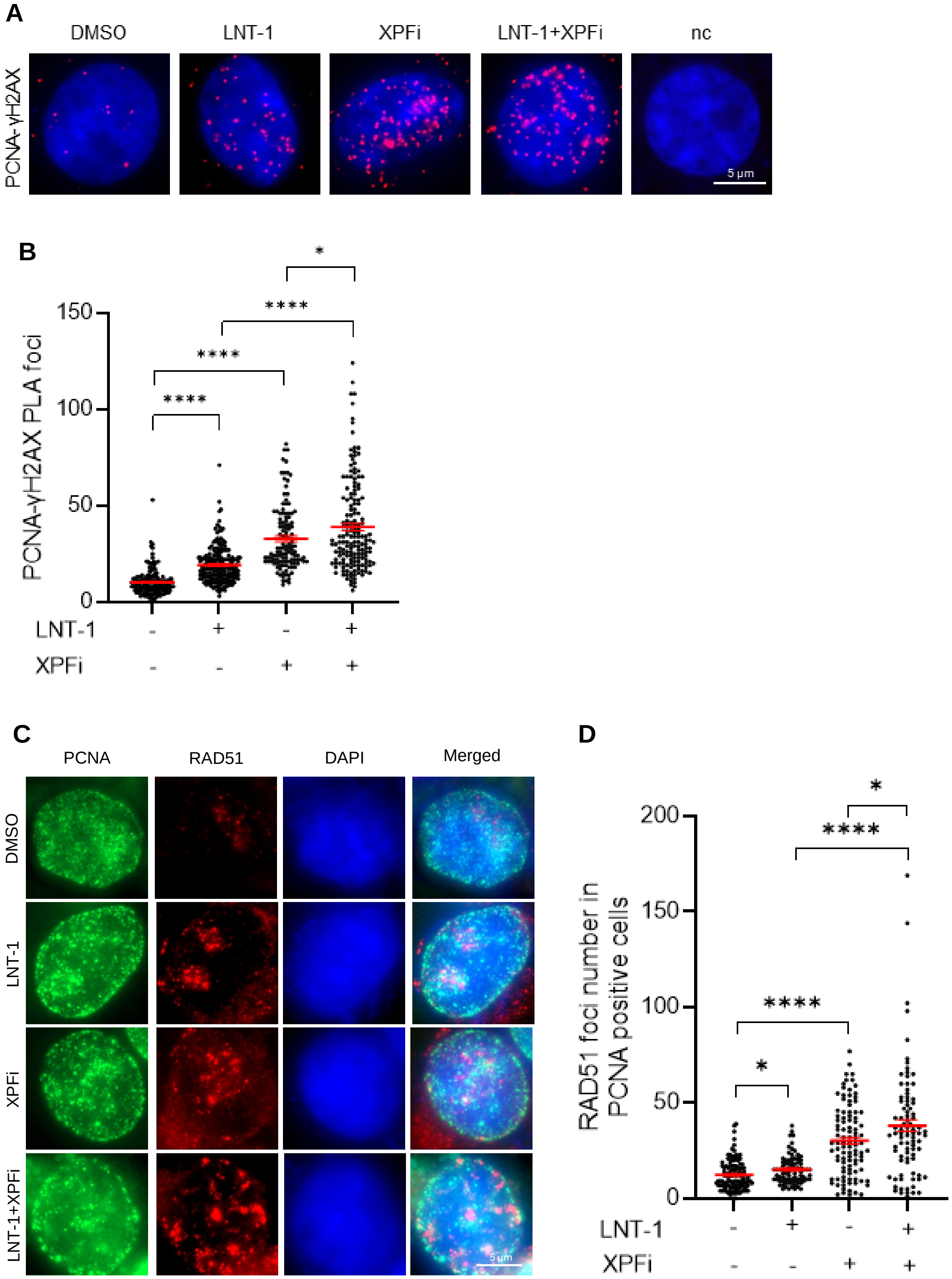

A
DMSO
LNT-1
XPFi
LNT-1+XPFi
nc
PCNA-γH2AX
5 μm
B
PCNA-γH2AX PLA foci
150
100
50
0
*
****
****
****
LNT-1
XPFi
C
PCNA
RAD51
DAPI
Merged
DMSO
LNT-1
XPFi
LNT-1+XPFi
5 μm
D
RAD51 foci number in PCNA positive cells
200
150
100
50
0
*
****
****
*
LNT-1
XPFi

Supplementary Figure S11: DNA replication-associated damage induced by FEN1 and XPF inhibitors. (A) Representative microscope images of PLA performed in MDA-MB-231 cells treated with DMSO/ FEN1i (LNT-1)/ XPFi; (B) Quantification of the PCNA- γH2AX PLA foci number in MDA-MB-231 cells. Cells were treated with DMSO/ FEN1i LNT-1 (10 μM)/ XPFi (10 μM) for 16hr. Single-antibody negative controls were performed in LNT-1 (10 μM) treatment cells and showed minimal background signal. Data represent mean ± SEM from ≥100 cells per condition. * $p < 0.05$, ****$P < 0.0001$. P values are calculated by the student's t-test; **(C)** Representative microscope images of RAD51-PCNA co-IF staining in MDA-MB-231 cells treated with DMSO, FEN1i LNT-1 or XPFi; **(D)** Quantification of the RAD51 foci number in per PCNA positive cell. MDA-MB-231 cells were treated with DMSO/ FEN1i LNT-1 (10 μM)/ XPFi (10 μM) for 16hr. Data represent mean ± SEM from ≥100 cells per condition. * $p < 0.05$,**** $p < 0.0001$. P values were calculated with the student t-test.

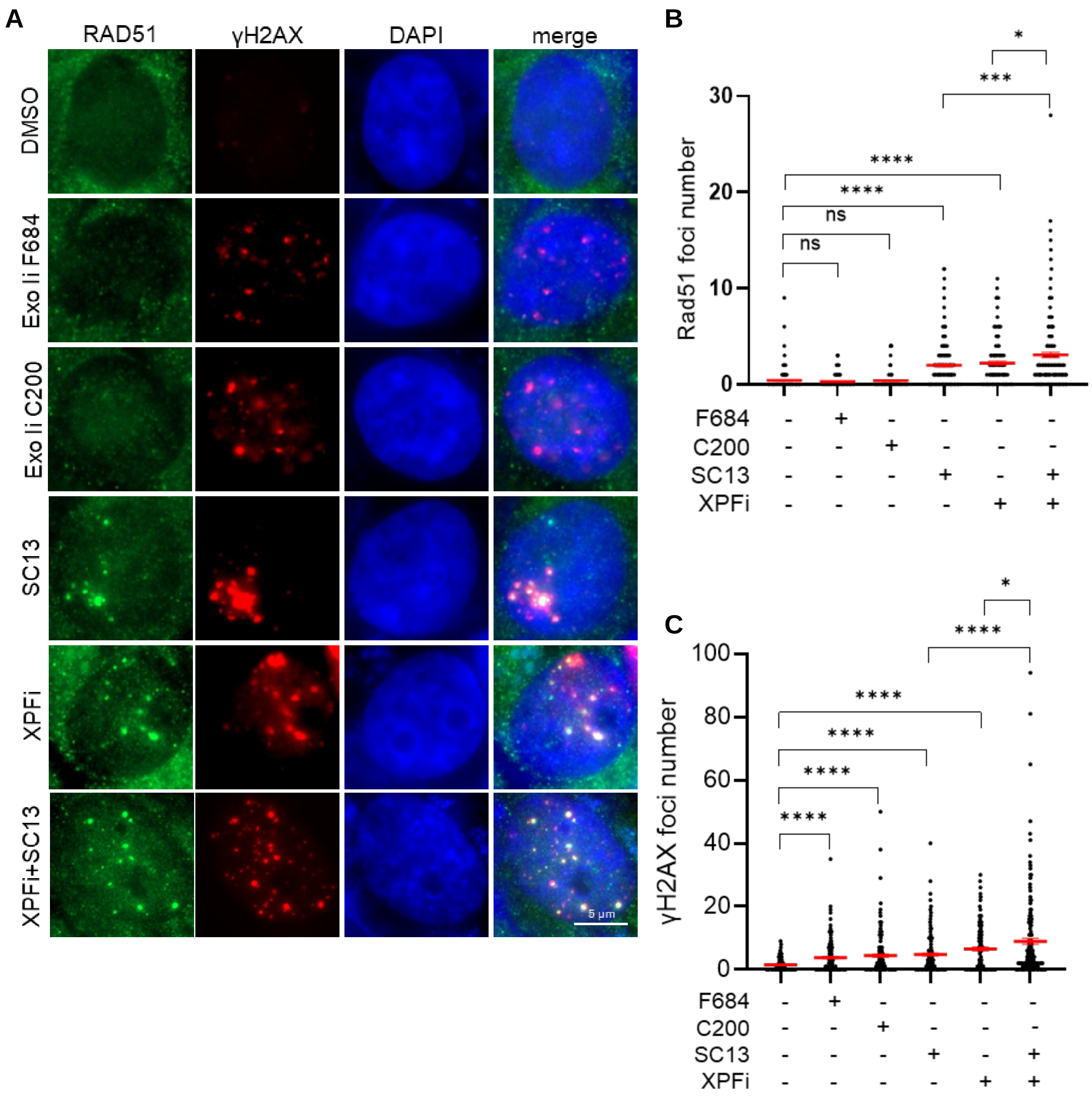

Supplementary Figure S12: γH2AX and RAD51 co-immunofluorescence (co-IF) staining in MDA-MB-231 cells treated with EXO1 inhibitor or FEN1 inhibitor SC13. (A) Representative microscope images of γH2AX and RAD51 IF in WT and *Ercc4-/-* MDA-MB-231 cells treated with DMSO/ EXO1 inhibitor F684/ EXO1 inhibitor C200/ FEN1 inhibitor SC13/ XPF inhibitor; Quantification of γH2AX **(B)** and RAD51 **(C)** foci number per cell in IF staining. MDA-MB-231 cells were treated with DMSO/ EXO1 inhibitor F684 (10 μM)/ EXO1 inhibitor C200 (10 μM)/ FEN1 inhibitor SC-13 (10 μM)/ XPF inhibitor (10 μM) for 16hr. Data represents mean ± SEM from ≥100 cells per condition. *P<0.05, ***P < 0.001, ****P < 0.0001. P values from Student t-test.

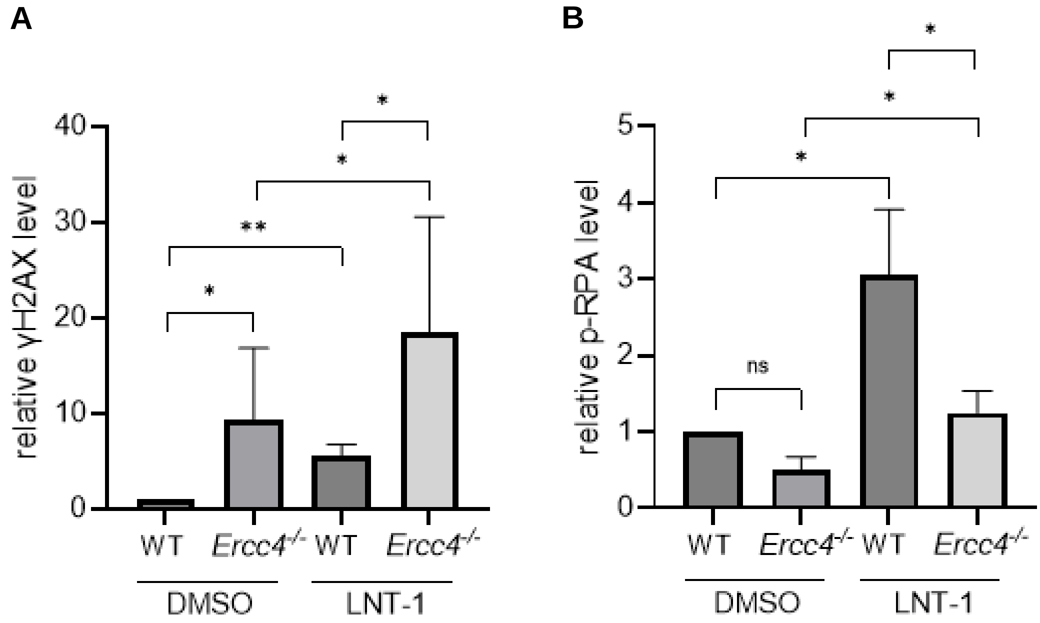

Supplementary Figure S13: Quantification of immunoblot analysis showing increased γH2AX and p-RPA in XPF deficient cells. (A) Quantification of γH2AX protein levels shown in figure4 I. Band intensities were quantified using ImageJ, normalized to GAPDH, and expressed relative to WT cells. Data represent mean ± SEM from three independent experiments; **(B)** Quantification of p-RPA protein levels shown in figure4 I. Band intensities were quantified using ImageJ, normalized to GAPDH, and expressed relative to WT cells. Data represent mean ± SEM from three independent experiments. *P<0.05, **P < 0.01. p values are calculated by the student's t-test.

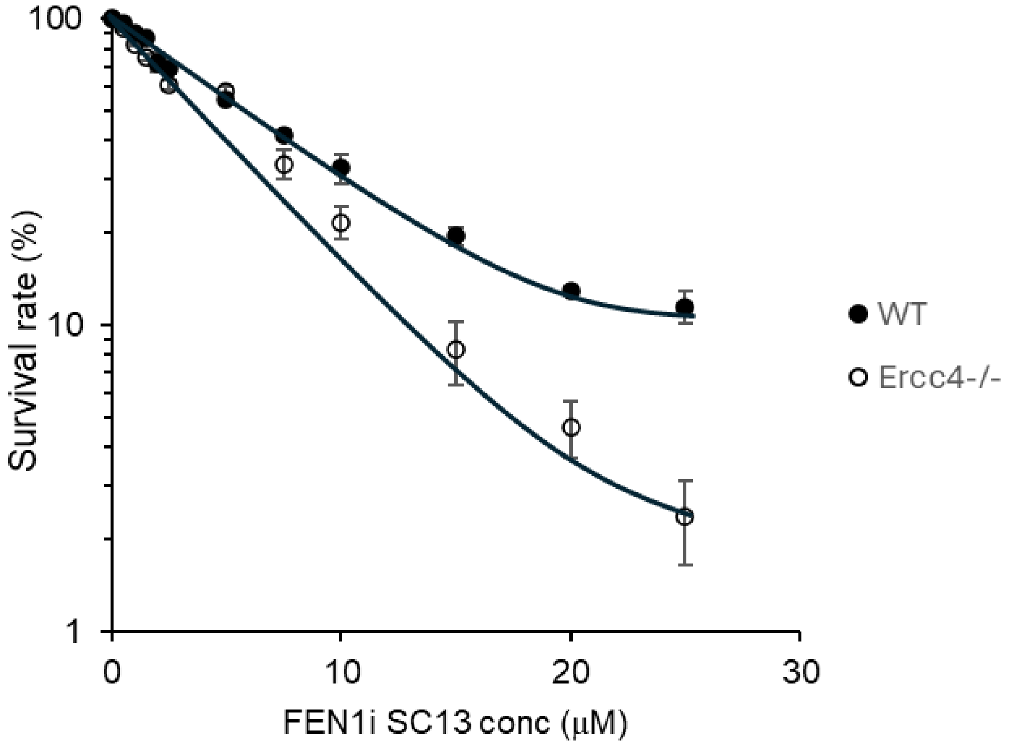

Supplementary Figure S14: Synergistic effects of FEN1i SC13 in killing MDA-MB-231 cells. Sensitivity of WT or Ercc4-/- MDA-MB-231 cells to FEN1i SC13. Cells were treated with varying concentrations of FEN1i SC13 for 4 days, and the viable cells were counted. The survival rate of each treatment was calculated relative to untreated control. Data represents mean ± SD, n = 3 independent treatments.

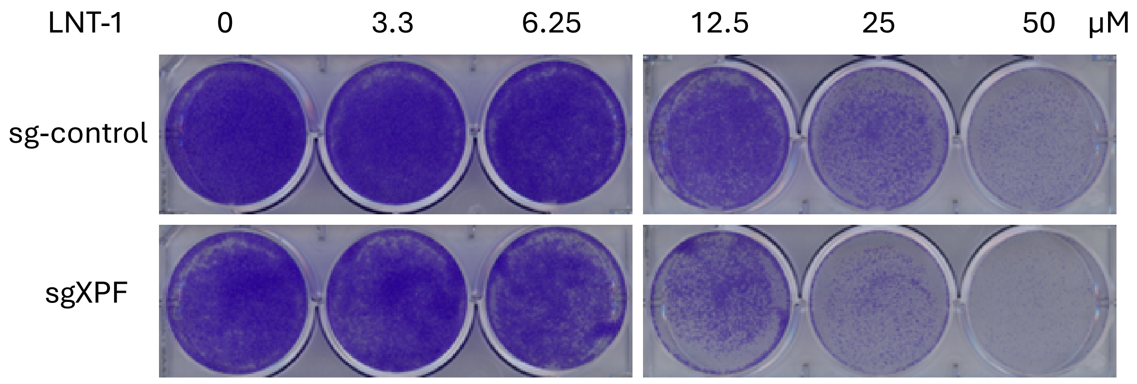

Supplementary Figure S15: Cell viability assay in WT (sg-control) or ERCC4-/- (sgXPF) MDA-MB-231 cells. WT or ERCC4-/- cells were cultured in DMEM containing varying concentrations of FEN1i (LNT-1) for 10 days. Viable cells were stained with Crystal-violet solution.
